# Case Report: The Conundrum of What to Pick? Antibiotic Susceptibility Variability in *Burkholderia cenocepacia* in Cystic Fibrosis: Implications for Antibiotic Susceptibility Testing and Treatment

**DOI:** 10.3389/bjbs.2024.12749

**Published:** 2024-06-04

**Authors:** John E. Moore, John McCaughan, Jacqueline C. Rendall, Beverley C. Millar

**Affiliations:** ^1^ Laboratory for Disinfection and Pathogen Elimination Studies, Northern Ireland Public Health Laboratory, Belfast City Hospital, Belfast, Northern Ireland, United Kingdom; ^2^ Northern Ireland Regional Adult Cystic Fibrosis Centre, Belfast City Hospital, Belfast, Northern Ireland, United Kingdom; ^3^ Department of Medical Microbiology, The Royal Group of Hospitals, Belfast, Northern Ireland, United Kingdom

**Keywords:** antibiotic treatment, antibiotic susceptibility, *Burkholderia cenocepacia*, cystic fibrosis, CF, biomedical science, microbiology

## Abstract

Within cystic fibrosis microbiology, there is often mismatch between the antibiotic susceptibility result of an isolated bacterial pathogen and the clinical outcome, when the patient is treated with the same antibiotic. The reasoning for this remains largely elusive. Antibiotic susceptibility to four antibiotics (ceftazidime, meropenem, minocycline and trimethoprim-sulfamethoxazole) was determined in consecutive isolates (*n* = 11) from an adult cystic fibrosis patient, over a 63 month period. Each isolate displayed its own unique resistotype. The first isolate was sensitive to all four antibiotics, in accordance with Clinical and Laboratory Standards Institute methodology and interpretative criteria. Resistance was first detected at four months, showing resistance to ceftazidime and meropenen and intermediate resistance to minocycline and trimethoprim-sulfamethoxazole. Pan resistance was first detected at 18 months (resistotype IV), with three resistotypes (I, II and III) preceding this complete resistotype. The bacterium continued to display further antibiotic susceptibility heterogeneity for the next 45 months, with the description of an additional seven resistotypes (resistotypes V–XI). The Relative Resistance Index of this bacterium over the 63 month period showed no relationship between the development of antibiotic resistance and time. Adoption of mathematical modelling employing multinomial distribution demonstrated that large numbers of individual colony picks (>40/sputum), would be required to be 78% confident of capturing all 11 resistotypes present. Such a requirement for large numbers of colony picks combined with antibiotic susceptibility-related methodological problems creates a conundrum in biomedical science practice, in providing a robust assay that will capture antibiotic susceptibility variation, be pragmatic and cost-effective to deliver as a pathology service, but have the reliability to help clinicians select appropriate antibiotics for their patients. This study represents an advance in biomedical science as it demonstrates potential variability in antibiotic susceptibility testing with *Burkholderia cenocepacia*. Respiratory physicians and paediatricians need to be made aware of such variation by biomedical scientists at the bench, so that clinicians can contextualise the significance of the reported susceptibility result, when selecting appropriate antibiotics for their cystic fibrosis patient. Furthermore, consideration needs to be given in providing additional guidance on the laboratory report to highlight this heterogeneity to emphasise the potential for misalignment between susceptibility result and clinical outcome.

## Introduction

There are approximately 1,315 people registered in Ireland with cystic fibrosis (CF) [1] and 10,908 people with CF registered in the United Kingdom [2]. These people are likely to develop lung infections of an acute, intermittent or chronic nature, sometime during their disease journey, which will require clinical intervention in the form of tablet, nebulised or intravenous antibiotics [3]. Lung infections develop in the person with CF due to the underlying pathophysiology of this autosomal recessive life-limiting genetic rare disease, due to an inability of the patient to clear respiratory secretions, often resulting in entrapment of environmental organisms, including bacteria and fungi, with the manifestation of lung infections [3]. For a seminal review of the pathophysiology of CF, please see Shteinberg et al. review [4].

*Burkholderia cenocepacia* is a clinically significant Gram-negative pathogen isolated from the lungs of people with CF, associated with increased morbidity and mortality [5]. Traditionally, *Burkholderia cenocepacia* organisms in CF have represented some of the most challenging clinical dilemmas in antibiotic management of CF, due to high levels of antibiotic resistance. In Ireland, antibiotic resistance in these organisms has been explained, at least in part, by the presence of class 1 integrons with recombined gene cassettes containing bla-OXA and aac(6′)-1a genes [6].

Antibiotic susceptibility (AS) testing of CF pathogens is important to help guide respiratory physicians and paediatricians in selecting appropriate antibiotics to treat their patients. Antibiotics may be employed in three clinical scenarios, namely, 1) to eradicate CF pathogens on their first isolation, 2) to suppress CF pathogens during chronic infection and 3) to treat serious infections, as part of pulmonary exacerbations. Unlike other infections within clinical microbiology, where there is good alignment between the AS result and the clinical outcome of the patient, CF microbiology has suffered from a relative mismatch between the AS result and the clinical outcome of the patient. For example, this may happen in two contexts, namely, 1) the AS result indicates sensitivity, the clinician treats with that antibiotic, but there is no clinical improvement in the patient and 2) the AS results indicates resistance, the clinician treats with that antibiotic and there is clinical improvement in the patient. Such mismatches have evolved to an extent, which has undermined the value of AS testing (AST) with CF clinicians, leading to some CF clinician abandoning AS testing and opting for empirical antibiotic treatment [7].

Such a situation needs to be revisited by biomedical scientists and microbiologists to help decipher what is going on to have lead to such a lack of confidence of AS testing of CF pathogens. One possibility is that there is greater variability in resistant types (resistotypes) that are present in the lung at any given time, which are difficult to detect phenotypically, due the organisms appearing visibly indistinguishable, leading to the reporting of a narrow and limited range of resistotypes.

To help quantify and add more tools to the AS testing arsenal, in this journal, we have developed the Relative Resistance Index (RRI) [8]. RRI is a simple method where antibiotic susceptibility is quantified by allocating a score of 1 for sensitive, 2 for intermediate resistance and 3 for resistant, for each antibiotic tested. The RRI is the mean value across all antibiotics tested for the isolate and enables quantitative monitoring of antibiotic resistance.

Therefore, the aim of this study was to examine the heterogeneity of resistant phenotypes of an isolate of *Burkholderia cenocepacia* in an adult CF patient over a 63 month period, by examining the antibiotic susceptibility of consecutive isolates to four antibiotics (ceftazidime, meropenem, minocycline and trimethoprim-sulfamethoxazole), in accordance with the Clinical and Laboratory Standards Institute (CLSI) testing methodology and interpretive criteria [9] and to consider the ramifications of antibiotic resistance heterogeneity for routine antibiotic susceptibility testing for the clinical microbiology laboratory and for treatment by the respiratory physician.

### Details of the Case

Antibiotic susceptibility tests were performed *in vitro* on a collection of *Burkholderia cenocepacia* isolates (*n* = 11), from an adult CF patient over a 63 month period from initial isolation, against the following four antibiotics: ceftazidime, meropenem, minocycline and trimethoprim-sulfamethoxazole. Antibiotic susceptibility testing was performed in accordance with the Clinical and Laboratory Standards Institute (CLSI) M100-ED33:2023 Performance Standards for Antimicrobial Susceptibility Testing [9], in terms of testing methodology and interpretive criteria. Resulting zone sizes (mm) to disks were recorded as either sensitive (S), intermediate (I) or resistant (R) [9]. Each isolate was categorised into a resistotype, based on the susceptibility to each of the four antibiotics.

The Relative Resistance Index (RRI) was calculated for each isolate, combining the individual susceptibility to each of the four antibiotics, where a mean score = 1, was complete sensitivity to all antibiotics tested and a mean score = 3, represented complete resistance to all antibiotics tested, as previously described [8]. The Relative Resistance Index (RRI) is a simple method where antibiotic susceptibility is quantified by allocating a score of 1 for sensitive, 2 for intermediate resistance and 3 for resistant, for each antibiotic tested. The RRI is the mean value across all antibiotics tested for the isolate and enables quantitative monitoring of antibiotic resistance.

**FIGURE 1:**
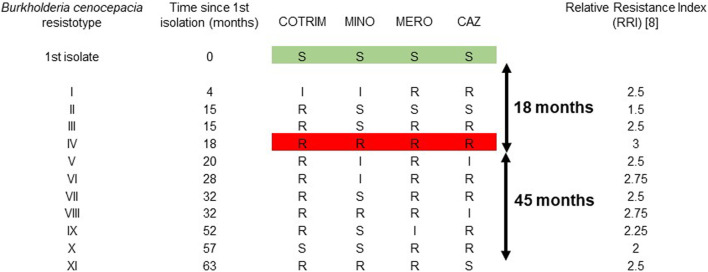
Description of 11 resistotypes of *Burkholderia cenocepacia* isolated from an adult CF patient over a 63 month period. The resistotype profiles are based on susceptibility to four antibiotics (ceftazidime, meropenem, minocycline and trimethoprim-sulfamethoxazole), in accordance with the Clinical and Laboratory Standards Institute (CLSI) testing methodology and interpretive criteria. S, sentive; I, intermediately resistant; R, resistant. CAZ, ceftazidime; COTRIM, trimethoprim-sulfamethoxazole; MERO, meropenem; MINO, minocycline.

Over the 63 month period, *B. cenocepacia* was isolated from all sputum examined routinely (*n* = 66), of which 11 isolates yielded a unique resistotype. These isolates were from sputum routinely collected both at routine out-patient clinics, as well as during in-patient stays, as part of the patient’s routine care. Strain characterisation/typing was performed which showed the isolates to have a rare phenotypic and molecular uniqueness, namely, cable pilus negative and mucoid, which was present throughout. These eleven isolates represented 11 different resistotypes (Figure 1). The first isolate (t = 0 month) was sensitive to all four antibiotics tested, in accordance with CLSI interpretative criteria. Resistance to antibiotics was first detected at 4 months, with an antibiogram showing resistance to ceftazidime and meroperen and intermediate resistance to minocycline and trimethoprim-sulfamethoxazole (R, R, I, I). Pan resistance was first detected at 18 months, with three resistotypes (resistotypes I, II and III) preceding the complete resistotype (resistotype IV). The bacterium continued to display further heterogeneity in its susceptibility profile, for the next 45 months, with the description of an additional seven resistotypes (resistotypes V–XI) (Figure 1). The Relative Resistance Index (RRI) of this bacterium over the 63 month period is shown (Figure 1). There was no relationship between the development of antibiotic resistance over time (*r* ^2^ = 0.0583).

## Discussion

Previously, Foweraker et al. demonstrated variability in the reporting of antibiotic susceptibility with *Pseudomonas aeruginosa* from CF aputum [10]. In their study, they showed the co-existence of several resistotypes in a single genotype of *P. aeruginosa*, even when the colony types appeared similar and further showed that the wide range of zone sizes in disc diffusion tests illustrated the variation in susceptibility of 48 colonies from one sputum sample. These authors concluded that the role of conventional antimicrobial susceptibility testing is questionable once *P. aeruginosa* chronically infects the cystic fibrosis lung, where several susceptibility patterns are seen, even within a morphotype and that routine susceptibility often underestimates resistance.

Hypermutation in CF pathogens is regarded as an important mechanism of adaptability of the organism during chronic infection, including a response to challenges to the organism’s persistence in the CF lung, including 1) natural survival pressures from the host immune system and 2) imposed survival pressure from antibiotics during patient treatment. Such hypermutational adaptation has been described for CF pathogens including *Pseudomonas aeruginosa* [11], *Staphylococcus aureus* [12] and *Stenotrophomonas maltophilia* [13] A study by Martina et al. showed that the distribution of hypermutator isolates amongst members of the *Burkholderia cepacia* complex, ranged from 0% with *B. vietnamiensis* to 31.3% in *B. cenocepacia* [14]. Interestingly, this study was unable to link the emergence of hypermutators with enhanced antibiotic resistance, with the exception of the fluoroquinolone, ciprofloxacin. Instead, these authors concluded that the high prevalence of hypermutators in *Burkholderia* organisms may be explained by their co-selection with other mutations involved in the bacterial pathoadaptability to the CF lung environment [14].

As part of the routine laboratory of workup of *Burkholderia* isolates, biomedical scientists will pick several morphologically similar colonies from the purity plate or non-selective plate, in order to prepare an inoculum for subsequent antibiotic susceptibility testing, in accordance with EUCAST testing methodology [15]. Where different morphotypes are visible, then this will be repeated with each visibly distinct morphotype, and the process repeated so that all visibly distinct morphotypes are covered. This practice will therefore obtain an antibiotic susceptibility result for each morphotype present. Foweraker et al. data showed that the picking of similar morphotypes of *P. aeruginosa* with different susceptibilities can be a chance event, as there may be several hundred different colonies on the plate of the same morphotype, but with several different susceptibility profiles within the one morphotype [10]. Therefore, careful considerations needs to be given to what is picked of the plate for susceptibility testing and how representative these colonies are, in relation to the overall population on the plate? Foweraker et al study demonstrates that the more we pick from the plate, the more representative the range of susceptibility will be detected. However, from a busy hospital microbiology laboratory’s perspective, it would not be pragmatic nor cost effective to pursue susceptibility testing in this manner. This, therefore, creates the challenge of what resources to invest in susceptibility testing to achieve a reliable and representative antibiogram, that is meaningful and adds value to the physician in helping to decide upon what antibiotic(s) to prescribe.

**FIGURE 2:**
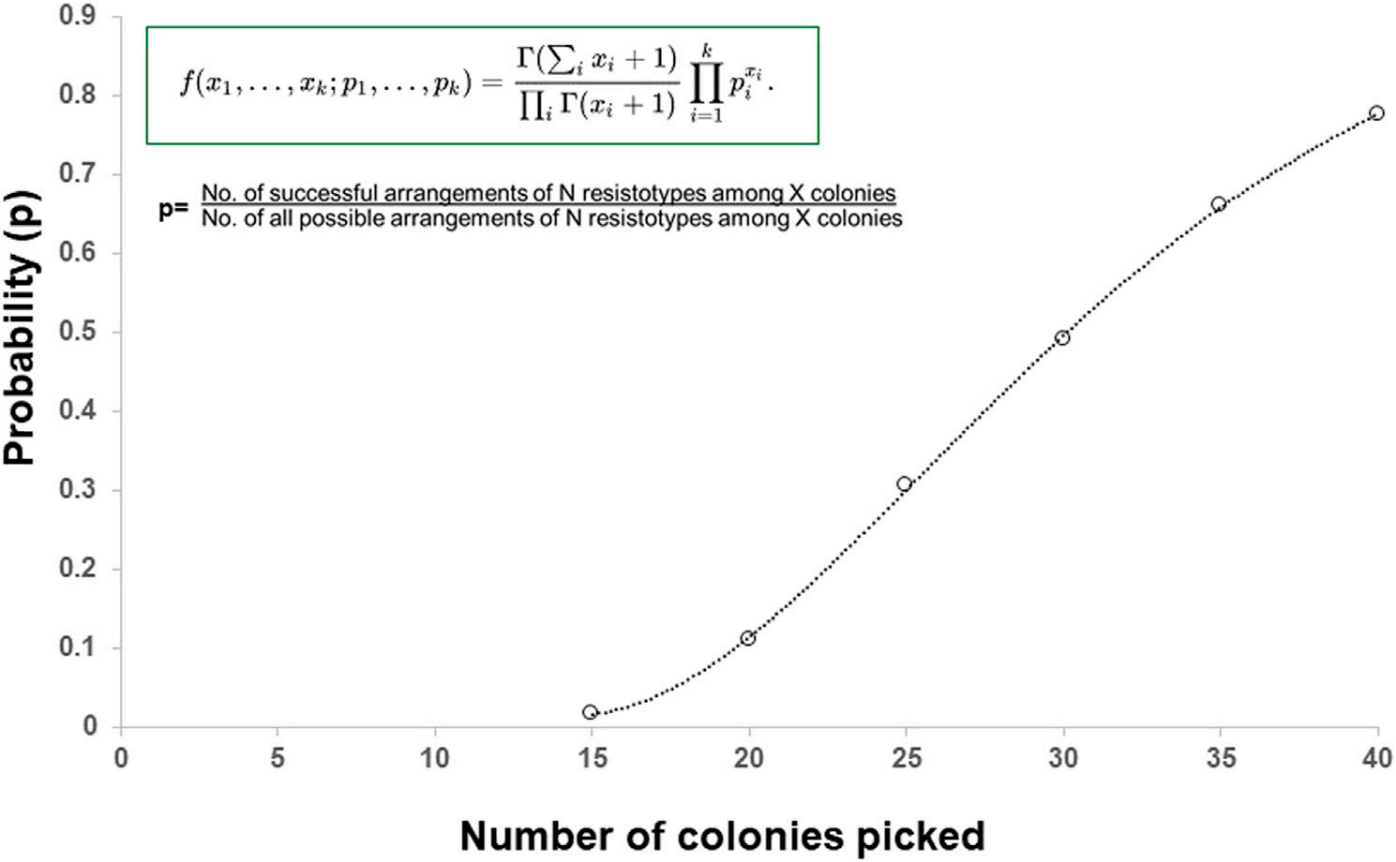
The probability (P) of picking at least one colony of each 11 resistotypes, present at equal frequencies in an infinite population, in a sample of 0–40 colonies randomly picked from the population.

In our case, the isolate of *B. cenocepacia* yielded 11 resistotypes, from four antibiotics with three possible susceptibility outcomes per antibiotic, namely, S, I or R. Mathematically, there are a total of 81 possible permutations of these antibiotics/susceptibilities (i.e., 3 × 3 × 3 × 3 = 3^4^ = 81), of which our studies had already identified 11/81 (13.6%) of these resistotypes. One rational approach to help estimate the number of colonies to pick from the plate for susceptibility testing to ensure the equal likelihood of randomly all resistotypes would be to employ a mathematical solution. Previously, such a strategy has been applied to picking morphologically indistinguishable serogroups of *E. coli* from animal and human faecal material by Hedges et al. [16]. Here, the authors based their statistical model on a multinomial distribution, where faecal coliforms constituted an infinitely large population in which N serotypes were present at frequencies p, p1, p2,p3,….pi, … … pN. It also assumed that morphologically indistinguishable colonies selected from a culture plate for serotyping were equivalent to a “random sample” drawn from the population. Adoption of this statistical approach and multinomial distribution algorithm to our study of 11 colonially indistinguishable resistotypes of *B. cenocepacia* (Figure 2), shows, for example, that 15 individual colonies are picked from a plate of a known population of 11 resistotypes, the probability of equally detecting all resistotypes is 0.018 (1.8%), whereas if 40 individual colonies are picked, the probability rises to 0.776 (77.6%). Adoption of such a mathematical approach offers a structured way of deciding on the required numbers of colonies to pick, based on the expected total number of resistotypes present in the sputum. One potential caveat to this approach however is that this model relies on equal frequencies of the phenotypes present. Presently, we are unsure if the resistotypes present in CF sputum are present in equal frequencies. Nevertheless, such algorithms may aid in the planning of efficient sampling programmes for determining most or all resistotypes present in CF sputum.

Other groups have offered up alternative solutions to address this issue of antibiotic susceptibility variability and how best to efficiently determine this. Zeboud et al. described the Direct sputum antimicrobial susceptibility testing (DSST) method, which involves e-tests being applied directly to a plate inoculated with 1/20 dilution of CF sputum and concluded that the DSST method was an efficient and easy antibiotic susceptibility testing method [17]. A similar approach would be to flood the primary non-selective plate (Columbia Blood Agar plate) to capture all organisms, however EUCAST methodology advises against this on health and safety grounds because pipetting and decanting high concentrations of organisms in suspensions onto the surface of plates, and subsequent removal, carries a high risk of production of aerosols and splashing [15]. Additionally, flooding tends to produce higher density of microorganisms over the agar surface when compared with swabbing [15]. Furthermore, having navigated through a rationale for what gets picked and what does not, the reliably of antibiotic susceptibility testing of *Burkholderia* isolates is complicated [18]. Antimicrobial susceptibility of *Burkholderia cepacia* complex (BCC) organisms, including *Burkholderia cenocepacia*, is problematic for a variety of reasons, as detailed by EUCAST in Table 1.

**TABLE 1:** Antimicrobial susceptibility testing of *Burkholderia cepacia* complex (BCC).

Antimicrobial susceptibility testing
It is not currently possible to establish minimum inhibitory concentration (MIC) breakpoints for BCC organisms as:
• There is no evidence to describe a relationship between MIC and outcome
• BCC is frequently part of a mixed infection
• The MIC distribution of BCC for relevant antimicrobials is wide and encompasses the non-species related pharmacodynamic breakpoint. Therefore the epidemiological cut-off value cannot be used to define the wild-type population as either susceptibile or resistant
Susceptibility testing methodology is problematic:
• MIC determination by the ISO broth microdilution (BMD) method with Mueller-Hinton broth yields reproducible results
• MIC determination by the gradient strip method is less reproducible than BMD
• The correlation between the MIC by the ISO BMD method and disk diffusion zone diameters is poor when tested by EUCAST (on Mueller-Hinton agar) or British Society for Antimicrobial Chemotherapy (BSAC) (on Isosensitest agar) methods

Taken from https://www.eucast.org/fileadmin/src/media/PDFs/EUCAST_files/General_documents/BCC_susceptibility_testing_130719.pdf.

These data have been produced in part under ECDC service contracts and made available by EUCAST at no cost to the user and can be accessed on the EUCAST website. The views and opinions expressed are those of EUCAST at a given point in time. EUCAST recommendations are frequently updated in the latest versions.

Antimicrobial resistance is a major problem in cystic fibrosis [19]. There is controversy that the *in vitro* antibiotic susceptibility of isolates has little impact on clinical outcome. Traditionally, clinicians are informed on the AST of isolates obtained from their patients’ sputum and make a clinical management decision on antibiotic choice, based on some form of such AST results. However, there is much debate as to the value of employing AST and how this impacts clinical outcomes. Historically, a study from the UK in 2005 showed that the CF lung may be chronically colonised with several phenotypes of PA, each with their unique susceptibility pattern, even within a single morphotype and concluded that routine antibiotic susceptibility testing is not reproducible and underestimates resistance [10]. Previously, VanDevanter et al. examined the relationship between antibiotic susceptibility and clinical parameters, including change in weight and lung function (ppFEV1) and were unable to demonstrate superior clinical improvements based on antibiotic susceptibility guided therapy. These authors concluded that AST may be of little utility in choosing antimicrobial therapy for *Pseudomonas aeruginosa*-related pulmonary exacerbations [20]. One possibility for such poor alignment between AST results and clinical outcome may be inadequate reporting of the diversity of all resistance types at the laboratory level, thereby placing more importance in developing and adopting suitable AST methodologies that address such AMR diversity, to help guide appropriate antibiotic prescribing.

In the current study, *B. cenocepacia* isolates showed increased resistance over time, suggesting that exposure to antibiotics may possibly drive antimicrobial resistance (AMR) development. Whilst it was not the aim of this case report, it would be interesting in future studies to attempt to correlate antibiotic usage with AMR development, with a larger cohort of CF patients, examining several organisms and involving several classes of antibiotics.

For this heterogeneity of resistance types in *B. cenocepacia*, antibiotic susceptibility testing of this species still remains problematic and a conundrum, which ultimately agrees with the EUCAST guidelines and lack of established breakpoints. The continued absence of such breakpoints and clarity from antibiotic susceptibility guidelines creates a dilemma for the CF clinician, when attempting to pick optimal antibiotics for treatment. Therefore, we hope that this case report re-ignites discussion amongst biomedical scientists and other key stakeholders, towards describing an optimal method for reliable and robust antibiotic susceptibility testing of this problematic pathogen in CF.

This study represents an advance in biomedical science as it demonstrates potential variability in AST with *Burkholderia cenocepacia*. Respiratory physicians and paediatricians need to be made aware of such variation by biomedical scientists at the bench, so that clinicians can contextualise the significance of the reported susceptibility result, when selecting appropriate antibiotics for their CF patient. Furthermore, consideration needs to be given in providing additional guidance on the laboratory report to highlight this heterogeneity to emphasise the potential for misalignment between susceptibility result and clinical outcome.

## Data Availability

The original contributions presented in the study are included in the article/supplementary material, further inquiries can be directed to the corresponding author.
